# Clinical and psychological features of the brain organization of mental activity in children with autistic disorders

**DOI:** 10.1192/j.eurpsy.2022.1558

**Published:** 2022-09-01

**Authors:** A. Pustovaya, E. Gutkevich, O. Shushpanova

**Affiliations:** 1 Tomsk state University, Faculty Of Psychology, Tomsk, Russian Federation; 2 Tomsk national research medical center Russian Academy of Sciences, Department Of Endogenous Disorders, Tomsk, Russian Federation; 3 Mental Health Research Centre, Department Of Childhood Psychiatry, Moscow, Russian Federation

## Abstract

**Introduction:**

Autism spectrum disorders (ASD) – nosological continuum of genetically and clinically heterogeneous mental disorders united by complex violation of mental development, social interaction and behavior.

**Objectives:**

Identify the features of brain functioning in children with ASD.

**Methods:**

Neuropsychological diagnostics (Zh. Glozman), results acoustic brainstem evoked potentials (ABEP), mathematical methods. The study involved 48 children with diagnoses (ICD-10): F84. 0, F84. 1, F84.5, aged 3 to 8 years (average age 4.18 years).

**Results:**

Neuropsychological examination revealed the main Indices of the functioning of brain blocks: the Index of activation and energy components of activity (I Index), the Index of the right-hemisphere holistic strategy of information processing (Index II-rights), the Index of the left-hemisphere analytical strategy of information processing (Index II-left), the Index of programming, regulation and control of activity (III Index). Then the children were divided into groups (p<0.001): 1 group – 10 children with high I Index (W = -6.03); 2 – 20 children with high Index II-rights (W = -5.74); 3 – 18 children with high III Index (W = -2.32). Correlation analysis showed: for group 1 difficulties in the perception of auditory information are characteristic; for group 2 – reduced level of control over the course of mental activity, difficulties in automating thinking and speech, coordination of movements; for group 3 – relationship is not manifested.

**Conclusions:**

Phonemic perception causes the greatest difficulties in children of group 1. Children of group 2 are characterized by difficulties in the formation of motor speech skills, a decrease in voluntary activity and control over it.

**Disclosure:**

No significant relationships.

